# Mitochondrial-to-nuclear communications through multiple routes regulate cardiomyocyte proliferation

**DOI:** 10.1186/s13619-024-00186-x

**Published:** 2024-01-31

**Authors:** Xinhang Li, Yalin Zhu, Pilar Ruiz-Lozano, Ke Wei

**Affiliations:** 1 Institute for Regenerative Medicine, Shanghai East Hospital, Shanghai Institute of Stem Cell Research and Clinical Translation, Shanghai Key Laboratory of Signaling and Disease Research, Frontier Science Center for Stem Cell Research, School of Life Sciences and Technology, Tongji University, Shanghai, 200092 P.R. China; 2 National Heart and Lung Institute, Imperial College London, London, UK; 3 Regencor Inc., 733 Industrial Road, San Carlos, CA 94070 USA

**Keywords:** Mitochondria, Cardiomyocyte, Proliferation, FAO, Cpt1b, αKG, H3k4me3, Mrps5, UPR^mt^, ATF4

## Abstract

The regenerative capacity of the adult mammalian heart remains a formidable challenge in biological research. Despite extensive investigations into the loss of regenerative potential during evolution and development, unlocking the mechanisms governing cardiomyocyte proliferation remains elusive. Two recent groundbreaking studies have provided fresh perspectives on mitochondrial-to-nuclear communication, shedding light on novel factors that regulate cardiomyocyte proliferation. The studies identified two mitochondrial processes, fatty acid oxidation and protein translation, as key players in restricting cardiomyocyte proliferation. Inhibition of these processes led to increased cell cycle activity in cardiomyocytes, mediated by reduction in H3k4me3 levels through accumulated α-ketoglutarate (αKG), and activation of the mitochondrial unfolded protein response (UPR^mt^), respectively. In this research highlight, we discuss the novel insights into mitochondrial-to-nuclear communication presented in these studies, the broad implications in cardiomyocyte biology and cardiovascular diseases, as well as the intriguing scientific questions inspired by the studies that may facilitate future investigations into the detailed molecular mechanisms of cardiomyocyte metabolism, proliferation, and mitochondrial-to-nuclear communications.

## Main text

After birth, mammalian cardiomyocytes undergo a profound maturation process characterized by a metabolic switch from glycolysis to fatty acid oxidation (FAO), hypertrophic growth with T-tubule formation, binucleation, and cell-cycle withdrawal. This maturation is accompanied by extensive alterations in both transcriptomic and epigenetic landscapes (Guo and Pu 2020). While decades of studies have identified multiple signaling pathways and molecules that regulate cardiomyocyte proliferation (Zheng et al. 2021) and placed cardiomyocyte cell-cycle withdrawal generally downstream of other postnatal changes such as metabolic switch (Cutie and Huang 2021), the intricate mechanisms underlying the limited proliferative capacity of postnatal cardiomyocytes have yet to be fully elucidated.

Notably, the heightened oxidative metabolism in postnatal cardiomyocytes, resulting in increased reactive oxygen species (ROS) generation, has been implicated in inducing DNA damage response and impeding the cell cycle (Puente et al. 2014). It has also been shown that disrupting the metabolic switch by deleting Pdk4 (Cardoso et al. 2020) and inhibiting succinate dehydrogenase (Bae et al. 2021) promote cardiomyocyte proliferation. Evolutionally, mitochondria have been implicated in multiple regeneration response across different species and organs (Zhao et al. 2023). However, despite the well-recognized role of ROS, which can be produced in the mitochondria and enters the nucleus, the mechanisms through which cardiomyocyte nuclei sense mitochondrial alterations and subsequently modulate transcription programs governing the cell cycle remain inadequately understood. Addressing this gap in knowledge is pivotal for comprehensively deciphering the regulatory networks that dictate cardiomyocyte proliferation postnatally.

Two recent works have looked into how mitochondria regulate cardiomyocyte proliferation with different perspectives (Gao et al. 2023; Li et al. 2023). Gao et al. employed a cardiomyocyte-specific heterozygous mutation of *Mrps5*, encoding a component of the mitochondrial ribosome (Gao et al. 2023). The resultant reduction in mitochondrial protein translation led to hyperplastic hearts, accompanied by augmented cardiomyocyte proliferation (Gao et al. 2023). Li et al., on the other hand, focused on disrupting mitochondrial FAO by inhibiting and deleting Cpt1b, a muscle-specific carnitine palmitoyltransferase responsible for transporting fatty acid into mitochondria (Li et al. 2023). This intervention similarly resulted in enlarged hearts and increased cardiomyocyte proliferation (Li et al. 2023). Remarkably, reducing Mrps5 and deleting Cpt1b not only led to hyperplastic hearts, but these mutations also promoted cardiac regeneration after myocardial infarction (MI) and ischemic-reperfusion injury, respectively (Gao et al. 2023; Li et al. 2023).

Both studies, despite converging on the outcome of cardiomyocyte proliferation through mitochondrial manipulation, took different approaches to dissect the molecular mechanisms linking mitochondria to cell cycle. Li et al. focused on metabolite analysis, revealing α-ketoglutarate (αKG) as the most significantly accumulated metabolite upon FAO blockage (Li et al. 2023). Given αKG’s essential role for many histone demethylases, the study investigated histone methylation and related enzymes, discovering that FAO blockage enhances Kdm5-mediated H3k4me3 demethylation in cardiomyocytes (Li et al. 2023). ChIP-Seq analysis unveiled a reduction in H3k4me3 on genes governing cardiomyocyte identity, suggesting a dedifferentiation towards a more immature and proliferative state (Li et al. 2023). On the other hand, Gao et al. delved into the mitochondrial unfolded protein response (UPR^mt^), a key mitochondrial-to-nuclear signaling pathway mediated by eIF2α phosphorylation and ATF4 activation (Gao et al. 2023). By reducing mitochondrial protein translation, the study demonstrated a robust activation of UPR^mt^, with direct binding of ATF4 to the promoters of cell division-related genes, such as Knl1, promoting cardiomyocyte proliferation (Gao et al. 2023). These elegant mechanistic studies revealed that mitochondria communicate with the nuclei through at least two distinct pathways—metabolites and UPR^mt^—to induce a pro-proliferative response in cardiomyocytes under stress or injury. This dual signaling suggests a multifaceted regulatory network between mitochondria and nuclei, highlighting the versatility of mitochondrial signals in governing cardiac regenerative responses.

Upon elucidating the molecular mechanisms orchestrating the pro-regenerative effects of *Mrps5* reduction and *Cpt1b* deletion, both studies sought to target these pathways with small molecules to induce cardiac regeneration after injury. It is not surprising that cardiomyocyte proliferation can be induced by αKG, which was shown to be drastically upregulated upon FAO blockage (Li et al. 2023). Intriguingly, Gao et al. adopted a distinctive approach by employing Doxycycline, a widely used molecule in reversibly inducible gene expression systems and known for inhibiting mitochondrial translation. By utilizing Doxycycline to induce UPR^mt^ in cardiomyocytes, the study observed enhanced proliferation and cardiac regeneration post-MI (Gao et al. 2023). The noteworthy outcome of cardiac regeneration, a challenging phenotype to achieve, through the application of a commonly used molecule such as Doxycycline raises important considerations for its usage in inducible gene expression systems. This finding underscores the need for caution in employing Doxycycline in such systems, aligning with recent recommendations for careful dosing and interpretation in experiments utilizing Doxycycline (De Boeck and Verfaillie 2021).

**Fig. 1:**
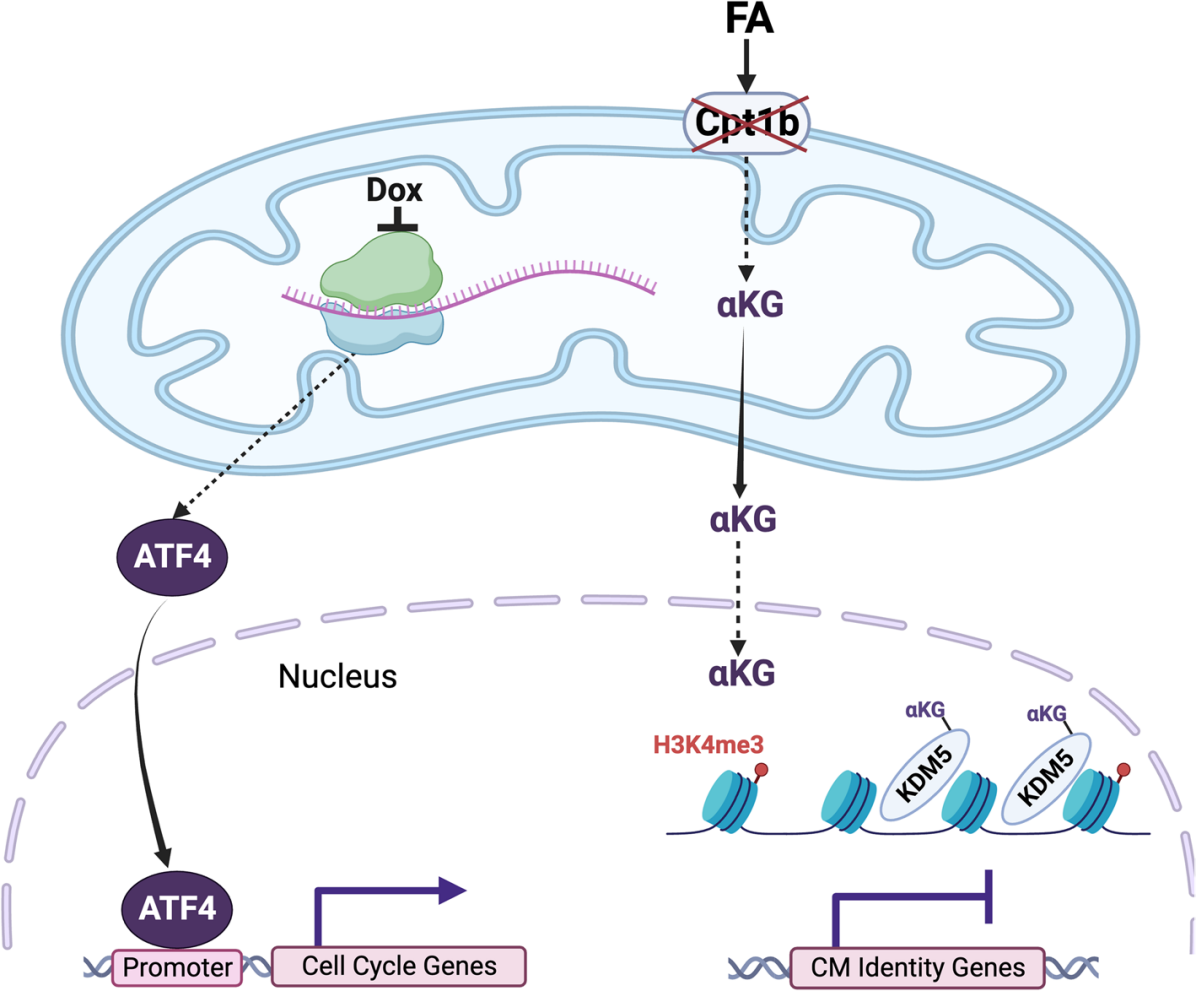
Disruption of mitochondrial protein translation and FAO metabolism promote cardiomyocyte proliferation through two distinct intermediates, UPR^mt^ mediated by ATF4, and H3k4me3 modifications through αKG and Kdm5, respectively. Dox: Doxycycline

Two distinct disruptions—mitochondrial protein translation and FAO metabolism—exert influence over the transcriptional programs governing cell cycle in the nuclei through two distinct intermediates, UPR^mt^ mediated by ATF4, and H3k4me3 modifications through αKG and Kdm5, respectively (Gao et al. 2023; Li et al. 2023) (Fig. 1). It is intriguing to understand whether these two pathways are cross-talking or even interdependent. It is plausible that UPR^mt^ may be triggered by FAO disruption, given its responsiveness to various mitochondrial stresses. Conversely, inhibiting mitochondrial translation might lead to metabolic alterations, influencing the concentrations of metabolites serving as substrates for histone modifications. In addition, other mechanisms briefly suggested in the studies, such as Hif1α activation by FAO inhibition (Li et al. 2023), which has been shown to regulate cardiomyocyte proliferation (Hashmi and Ahmad 2019), could also play a role in regenerative mitochondrial-to-nuclear communication. The potential interplay between UPR^mt^, histone modifications, and other pathways warrants further investigation into the synergistic and/or cooperative mechanisms through which signals from mitochondria converge to govern cardiomyocyte proliferation and cardiac regeneration.

The intriguing observation that the reduction in fatty acid entry or protein translation within cardiomyocyte mitochondria does not result in changes in cardiac function, despite an increase in cardiomyocyte numbers and an enlarged heart (Gao et al. 2023; Li et al. 2023), prompts several interesting hypotheses. One possibility is that cardiomyocytes are quite flexible in energy metabolism routes and can compensate for energy production when fatty acid and/or related proteins are not available. Indeed, Li et al. found that cardiomyocytes enhanced utilization of pyruvate and branched-chain amino acids as energy source to maintain a robust energy and functional output upon FAO blockage (Li et al. 2023). On the other hand, Gao et al. observed no change in metabolic gene expression and mitochondrial function upon *Mrps5* reduction (Gao et al. 2023), suggesting that cardiomyocytes may possibly synthesize proteins beyond the immediate requirements to establish a reservoir of metabolic machineries, potentially serving to meet fluctuations in demand. Alternatively, the increased numbers of cardiomyocytes, as observed in both studies, might act as a compensatory mechanism to offset potential reductions in metabolic and contractile functions at the individual cardiomyocyte level.

The activation of UPR^mt^ initiates downstream signals mediated by transcription factors such as CHOP, ATF4, and ATF5 (Anderson and Haynes 2020). Gao et al.’s finding revealed a unique response to mitochondrial translation inhibition, wherein ATF5 levels remained unchanged, CHOP levels decreased, and ATF4 was specifically activated (Gao et al. 2023). In addition, ATF4’s direct activation of genes regulating cell division was limited compared to its broader binding to over 4000 genes (Gao et al. 2023). The selective induction of ATF4, excluding other UPR^mt^ mediators, and its preferential activation of proliferation pose intriguing questions. The mechanism behind the specificity of ATF4 induction in response to reduced mitochondrial translation, and its ability to preferentially induce proliferation despite its broader transcriptional influence on various cellular processes, including metabolism in response to mitochondrial stress (Anderson and Haynes 2020), warrant further exploration. Similarly, the accumulation of αKG induced by FAO blockage serves as a substrate of multiple histone demethylases. The specificity observed in the reduction of H3k4me3, as opposed to other histone methylations in cardiomyocyte upon αKG accumulation, along with the enrichment of H3k4me3 reduction on genes governing cardiomyocyte identity, implies the involvement of epigenetic regulators with histone modification and chromosome position specificities in mediating the effect of FAO blockage. Unraveling the molecular mechanisms ensuring these specificities could provide valuable insights into the intricate regulatory network governing communication between mitochondria and nuclei, shedding light on the epigenetic nuances that dictate the outcomes of mitochondrial perturbations on cardiomyocyte biology.

## Conclusions

In summary, the two innovative studies addressed novel mechanisms regulating cardiomyocyte proliferation and converged on the mitochondrial-to-nuclear communications controlling cell cycle activity. Two distinct routes—UPR^mt^ mediated by ATF4, and H3k4me3 modifications through αKG and Kdm5—mediates effects of disruptions of mitochondrial protein translation and FAO blockage, respectively, to promote cardiomyocyte proliferation. There is no doubt these findings will significantly shape our understanding of the relationship between cardiac metabolism and regeneration, and have significant implications on both basic and clinical research on cardiac regeneration. Intriguing questions on the specificity of the mitochondrial-to-nuclear signals and the metabolic flexibility of the cardiomyocytes may lead to further investigations crucial for a comprehensive understanding of the communication between mitochondria and nuclei in cardiomyocytes. Such understanding will provide a foundation for targeted interventions, holding promise for unlocking the regenerative potential of the adult mammalian heart.

## Data Availability

Not applicable.

## Abbreviations

αKG: α-Ketoglutarate
UPR^mt^: Mitochondrial unfolded protein response
FAO: Fatty acid oxidation
ROS: Reactive oxygen species
